# Chinese herbal medicine Guizhi Fuling Formula for treatment of uterine fibroids: a systematic review of randomised clinical trials

**DOI:** 10.1186/1472-6882-14-2

**Published:** 2014-01-02

**Authors:** Ni-Ni Chen, Mei Han, Hong Yang, Guo-Yan Yang, Yu-Yi Wang, Xiao-Ke Wu, Jian-Ping Liu

**Affiliations:** 1 Center for Evidence-Based Chinese Medicine, Beijing University of Chinese Medicine, No. 11 Bei San Huan Dong Lu, Beijing, Chaoyang District, China; 2 Postgraduate College, Beijing University of Chinese Medicine, Beijing, China; 3 Department of Obstetrics and Gynecology, First Affiliated Hospital, Heilongjiang University of Chinese Medicine, Harbin, China

**Keywords:** Chinese herbal medicine, Guizhi Fuling Formula, Uterine fibroids, Systematic review, Meta-analysis, Randomised clinical trials

## Abstract

**Background:**

Guizhi Fuling Formula is widely applied for uterine fibroids in China. Many clinical trials are reported. This study assessed the efficacy and safety of Guizhi Fuling Formula for the treatment of uterine fibroids.

**Methods:**

PubMed, Cochrane CENTRAL, EMBASE, and four Chinese databases were searched through May 2013. We included randomised controlled trials (RCTs) that tested Guizhi Fuling Formula for uterine fibroids, compared with no intervention, placebo, pharmaceutical medication, or other Chinese patent medicines approved by the State Food and Drug Administration of China. Authors extracted data and assessed the quality independently. We applied RevMan 5.2.0 software to analyse data of included randomised trials.

**Results:**

A total of 38 RCTs involving 3816 participants were identified. The methodological quality of the included trials was generally poor. Meta-analyses demonstrated that Guizhi Fuling Formula plus mifepristone were more effective than mifepristone alone in reducing the volume of fibroids (in total volume of multiple fibroids, MD −19.41 cm^3^, 95% CI −28.68 to −10.14; in average volume of multiple fibroids, MD −1.00 cm^3^, 95% CI −1.23 to −0.76; in average volume of maximum fibroids, MD −3.35 cm^3^, 95% CI −4.84 to −1.87, I^2^ = 93%, random effects model). Guizhi Fuling Formula significantly improved symptoms of dysmenorrhea either when it was used alone (RR 2.27, 95% CI 1.04 to 4.97) or in combination with mifepristone (RR 2.35, 95% CI 1.15 to 4.82). No serious adverse events were reported.

**Conclusions:**

Guizhi Fuling Formula appears to have additional benefit based on mifepristone treatment in reducing volume of fibroids. However, due to high risk of bias of the trials, we could not draw confirmative conclusions on its benefit. Future clinical trials should be well-designed and avoid the issues that are identified in this study.

## Background

Uterine fibroids are the most common benign tumor of the female reproductive tract and occur in about 25% of all women of reproductive age [1] and up to 30–40% of women over age 40 [2]. It is estimated that 50% of fibroids are asymptomatic [3]. When fibroids are symptomatic, they present in a variety of ways such as menstrual problems, pain, pressure symptoms, and infertility. Current treatments include surgical approaches such as hysterectomy, myomectomy, uterine artery embolization (UAE) and magnetic resonance imaging–guided focused ultrasound surgery (MRgFUS), pharmacologic options such as hormonal therapies and gonadotropin-releasing hormone (GnRH) agonists [4]. However, surgery is associated with operative mortality and morbidity [1]. Medical therapy is similarly limited. GnRH agonists can relieve both bleeding and bulk-related symptoms but might cause significant menopausal side effects [5,6]. Progesterone antagonists, such as mifepristone and asoprisnil, seem to be effective in inducing fibroid regression without major adverse events. However, progesterone antagonists and other hormonal therapies that alter oestrogen and progesterone production or function may affect fertility [7]. So safer therapy is needed for uterine fibroids.

In China, traditional Chinese herbal medicine is a prevalent treatment for uterine fibroids. Guizhi Fuling Formula is one such common remedy. The formula was first described in *Essential Prescriptions from the Golden Cabinet* (*Jingui Yaolue*) by a famous Chinese doctor Zhang Zhongjing of the Han Dynasty (third century A.D.). Guizhi Fuling Formula consists of five herbs: *Ramulus Cinnamomi, Poria, Semen Persicae, Radix Paeoniae Rubra or Radix Paeoniae Alba, and Cortex Moutan*[8]. Its traditional effects (actions) are invigorating blood, dissolving stasis, and resolving masses [9]. Common preparations of the formula are pills, capsules, tablets, and decoctions. A bibliometrics study of modern literature analysing the names of diseases that are treated by Guizhi Fuling pills revealed that the highest frequency of traditional Chinese medicine (TCM) diseases was abdominal mass (*zheng jia*) and the highest frequency of western medicine diseases was uterine fibroids [10].

A Cochrane systematic review [11] assessing herbal preparations for uterine fibroids included ten clinical trials of Guizhi Fuling Formula. Results showed that the combination of Guizhi Fuling Formula and mifepristone was associated with a greater reduction in fibroid volume and in uterine size compared with mifepristone alone. In order to assess the efficacy and safety of Guizhi Fuling Formula for uterine fibroids, we conducted this study by comprehensively collecting and analysing randomised controlled trials (RCTs) on Guizhi Fuling Formula.

## Methods

The protocol of this systematic review was registered in the PROSPERO database (http://www.crd.york.ac.uk/PROSPERO/display_record.asp?ID=CRD42013003541).

### Search strategy

We searched three English electronic databases and four Chinese electronic databases from their inception through May 2013: PubMed, Cochrane CENTRAL, EMBASE, China National Knowledge Infrastructure (CNKI), Chinese Biomedicine (SinoMed), Chinese Scientific Journals Database (VIP), and WanFang Database. Conference proceedings and dissertations were also searched from CNKI and Wanfang databases for unpublished trials. The following search terms (or the Chinese equivalent for Chinese databases) were used individually or cross-linked and varied depending on which database was searched: “leiomyoma”, “leiomyomata”, “leiomyomas”, “fibroid”, “fibroids”, “uterine myoma”, “uterine myomas”, “uterine fibroid”, ‘uterine fibroids”, “uterine fibroma”, “uterine fibromas”, “Guizhi Fuling Formula”, “Guizhi Fuling decoction”, “Guizhi Fuling tang”, “Guizhi Fuling capsules”, “Guizhi Fuling pills”, “Guizhi Fuling tablets”, and “random”.

The specific search strategy of PubMed was as follows:

#1 Search ((((((((((leiomyoma[Title/Abstract]) OR leiomyomata[Title/Abstract]) OR leiomyomas[Title/Abstract]) OR fibroids[Title/Abstract]) OR fibroid[Title/Abstract]) OR uterine myoma[Title/Abstract]) OR uterine myomas[Title/Abstract]) OR uterine fibroids[Title/Abstract]) OR uterine fibroid[Title/Abstract]) OR uterine fibroma[Title/Abstract]) OR uterine fibromas[Title/Abstract]

#2 Search (((((guizhi fuling formula[Title/Abstract]) OR guizhi fuling decoction[Title/Abstract]) OR guizhi fuling tang[Title/Abstract]) OR guizhi fuling capsules[Title/Abstract]) OR guizhi fuling pills[Title/Abstract]) OR guizhi fuling tablets[Title/Abstract]

#3 Search random

#1 and #2 and #3

### Inclusion/exclusion criteria

#### Types of studies

We included randomised clinical trials (RCTs) assessing the beneficial effect and safety of Guizhi Fuling Formula for treating uterine fibroids. No language restriction was applied.

#### Types of participants

We did not limit the diagnostic criteria, but comprehensive criteria were accepted in terms of clinical symptoms, signs, plus confirmation by ultrasonography.

#### Types of interventions

Guizhi Fuling Formula in any preparations such as pills, capsules, decoctions, and tablets, orally taken, for the treatment of uterine fibroids were included. The treatment duration was no less than 30 days. Modified Guizhi Fuling Formula prescribed according to TCM syndrome differentiation was acceptable, and was defined by practitioners as adding no more than five herbs to the five original herbs, resulting in nearly the same actions as the original Guizhi Fuling Formula. The controls could be no intervention, placebo, medication, or other Chinese patent medicines approved by the State Food and Drug Administration of China. Trials testing combination of Guizhi Fuling Formula and medication compared with the same medication were also included.

#### Types of outcome measures

Primary outcomes were volume of uterine fibroids and symptom improvement: menstrual problems (extended, more frequent, heavy menstrual bleeding, vaginal bleeding at times other than menstruation, and anemia), abdominal mass, pressure symptoms (difficulty urinating or frequent urination, constipation), pain, leukorrhagia, infertility. Secondary outcomes were volume of uterus, recurrence rate, quality of life (QOL), and adverse events.

Redundant published trials, poor data authenticity trials (suspected fraud or plagiarism and information that could not be confirmed by authors when contacted), and trials with missing data not available from contacting the authors were excluded.

### Study selection and data extraction

Two authors (NNC, MH) conducted study selection and data extraction independently. Extracted information included: population, age and baseline characteristics; details of the intervention and control conditions; follow up time and outcome measures. Any disagreements were resolved through discussion with a third author (JPL).

### Assessment of risk of bias

Two authors (NNC, MH) independently assessed the quality of included trials using the Cochrane risk of bias tool [12]. The following items were assessed: random sequence generation, allocation concealment, blinding, incomplete outcome data, selective outcome reporting, and other bias. Sample size estimate, comparable baseline characteristic, inclusion and exclusion criteria were considered when we judged the other bias. Disagreements were resolved by discussion with a third author (JPL).

### Data analysis

We applied RevMan 5.2.0 software to conduct data analyses. We used risk ratios (RR) with 95% confidence intervals (CI) for binary outcomes or mean difference (MD) with 95% CI for continuous outcomes. When different measurement scales were used, standardised mean difference (SMD) analyses were performed. For cross-over trials, only the first phase outcome data were analysed. We applied fixed-effect model unless there was evidence of heterogeneity. Heterogeneity was assessed using both the Chi-squared test and the I-squared statistic with an I-squared value greater than 50% indicative of substantial heterogeneity. Funnel plots were generated to detect publication bias when more than ten trials were identified. When the necessary data were available, subgroup analysis was done for different parameters representing the volume of fibroids.

## Results

### Study search and selection

**Figure 1:**
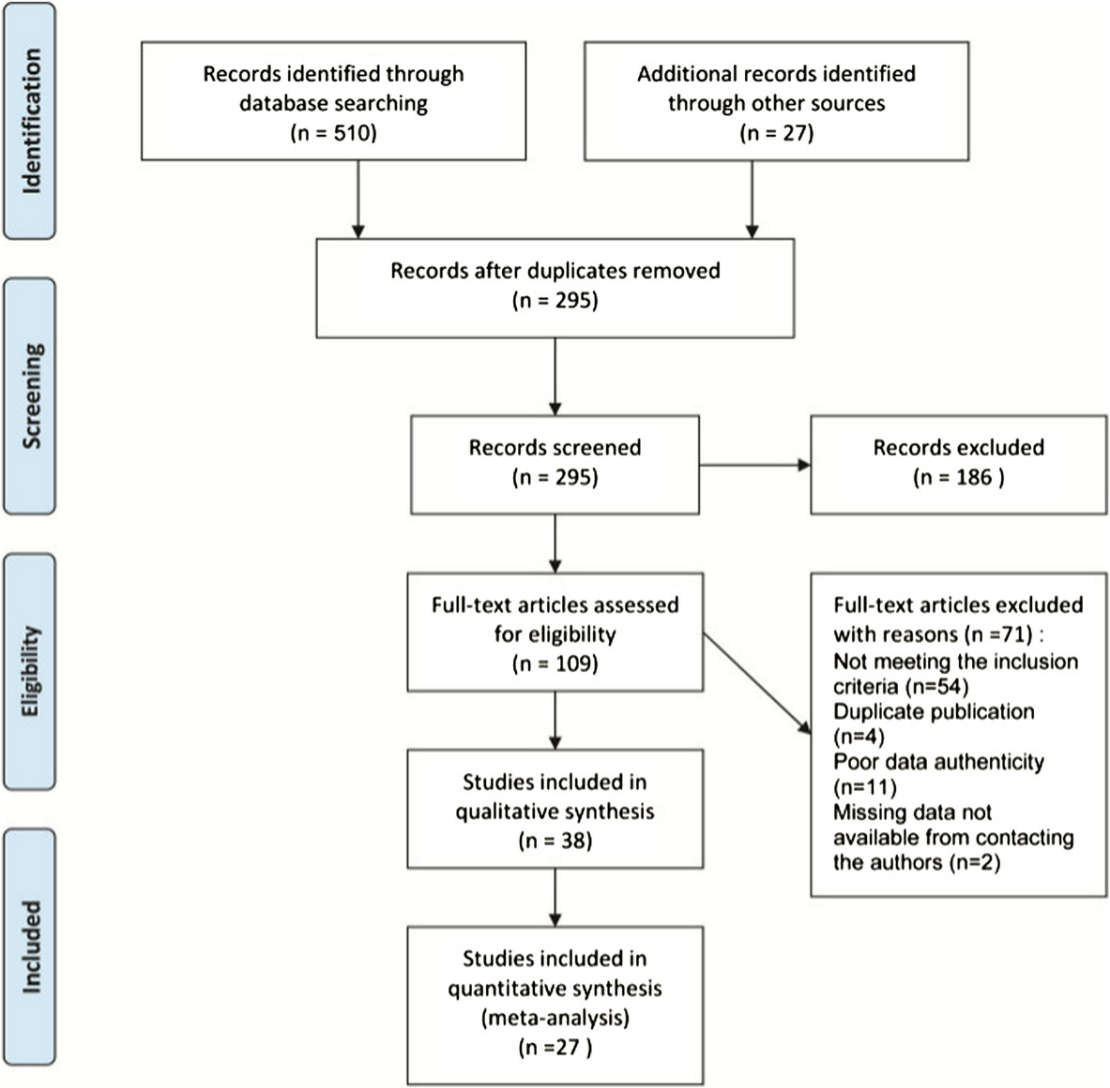
Flow chart.

We identified a total of 537 citations electronically, and we included 38 trials at last (Figure 1).

### Description of studies

Additional file 1 summarizes the characteristics of 38 included trials involving 3816 participants. All included trials were conducted in China and published in Chinese. Sample sizes varied from 39 to 195 participants, with an average of 100.42 participants per trial. All participants were diagnosed by clinical manifestation and ultrasonography. There was only one multicentre trial [13]. All of the trials had two arms, except two trials [14,15] that had three arms.

There were six trials comparing Guizhi Fuling Formula with western (conventional pharmaceutical) medicine; 30 trials comparing Guizhi Fuling Formula plus western medicine with western medicine; and three trials comparing Guizhi Fuling Formula with other Chinese patent medicines. One of the two trials [14,15] with three arms had two comparisons, which we counted twice. No trial applied placebo or no intervention as control. Preparations of Guizhi Fuling Formula were capsules or pills in all the included trials and were patent medicines approved by the State Food and Drug Administration of China.

For outcome measures, 34 trials reported fibroid volume and 12 trials reported uterus volume, and the volume was measured by ultrasonography. Five trials reported improvement of fibroid-related symptoms such as heavy menstrual bleeding, prolonged menstrual bleeding, vaginal bleeding at times other than menstruation, distention pain of the lower back and abdomen, and dysmenorrhea. Fifteen trials reported adverse events. Three trials though reported recurrence rate, but they did not define recurrence. No trial reported quality of life.

Of the 34 trials which reported fibroid volume, 21 trials reported different parameters to represent the volume of uterine fibroids. Among the 21 trials, 13 trials reported average volume of maximum fibroids, two trials reported total volume of multiple fibroids, four trials reported average volume of multiple fibroids, and two trials reported average diameter of fibroids. For example, a woman has three fibroids that are each 3 cm^3^, 4 cm^3^, and 11 cm^3^. If the volume of the largest fibroid is measured, the reported volume is 11 cm^3^; if total volume of multiple fibroids is measured, it is 18 cm^3^; if average volume of multiple fibroids is measured, it is 6 cm^3^. To confirm the parameters representing the volume of fibroids in the remaining trials that did not provide such information, we attempted to contact the authors of these trials and only the authors of two trials [16,17] provided detailed information. Volume of fibroids of 13 trials without clarified parameters was narratively synthesized.

### Methodological quality

**Figure 2:**
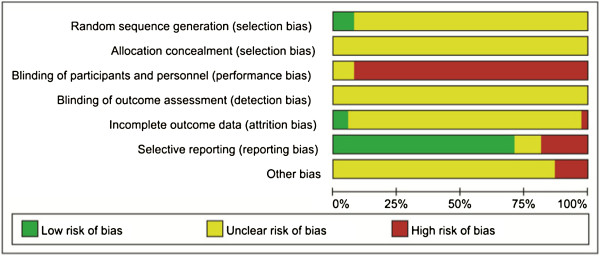
Methodological quality of included trials (summary of risk of bias).

All included trials were parallel-group randomised trials, but only three trials [13,18,19] (7.89%, 3/38) reported method of sequence generation (using random number table). No trial described allocation concealment or blinding. Two trials [13,20] (5.26%, 2/38) reported drop-out of participants, but intention-to-treat analysis was not used. For selective reporting, since the protocols of the 38 trials were all not accessible, we made our judgment by comparing the outcome measures mentioned in the method section with the reported outcomes in the results: 27 trials (71.05%, 27/38) reported all outcome measures described in the methods were evaluated as low risk; seven trials [20-26] (18.42%, 7/38) partially reported the outcomes in the results were evaluated as high risk, and four trials [17,27-29] (10.53%,4/38) were evaluated as unclear when no description of outcome measures in the methods. No trial had pre-trial sample size estimation. All trials were evaluated as high or unclear risk of bias (Figure 2; Additional file 2).

### Effect estimation

#### Volume of uterine fibroids

There were 34 trials that reported the volume of uterine fibroids. The effect of Guizhi Fuling Formula on volume of uterine fibroids are summarised in Table 1. We divided subgroups based on different parameters representing the volume of fibroids.

**Table 1:** Effect estimate of Guizhi Fuling Formula for volume of uterine fibroids

**Parameters representing the volume of fibroids**	**Study ID**	**MD [95% CI]**	***P *** **value**
***Guizhi Fuling Formula versus mifepristone***			
Average volume of maximum fibroids	Zhu YJ [30]	−1.43 [−3.23, 0.37]	0.12
Total volume of multiple fibroids	^a^Gao CR b [14]	−3.00 [−16.65, 10.65]	0.67
Average volume of multiple fibroids	Xiong DM [31]	0.50 [0.12, 0.88]	0.01
Volume of fibroids without clarified parameters	^b^Hu WH a [15]	0.19 [−0.12, 0.50]	0.22
	^c^Hu WH b [15]	−0.62 [−0.86,-0.38]	<0.00001
***Guizhi Fuling Formula plus mifepristone versus mifepristone***			
Average volume of maximum fibroids	Chen XJ [32]	−0.60 [−1.19, -0.01]	0.05
	Gu Y [33]	−0.83 [−1.50, -0.16]	0.02
	Teng MJ [34]	−19.36 [−24.33, -14.39]	<0.00001
	Wu YF [35]	−4.34 [−7.48, -1.20]	0.007
	Yang ZQ [36]	−0.58 [−1.16, -0.00]	0.05
	Yue Li [37]	−2.32 [−5.35, 0.71]	0.13
	Zhao YF [19]	−3.28 [−4.47, -2.09]	<0.00001
	Zhong GP [38]	−6.75 [−9.19, -4.31]	<0.00001
**Subgroup meta-analysis** (random, I^2^ = 93%)		−3.35 [−4.84, -1.87]	<0.00001
Total volume of multiple fibroids	^a^Gao CR a [14]	−15.00 [−28.95,-1.05]	0.04
	Lu HJ [39]	−22.90 [−35.31,-10.49]	0.0003
**Subgroup meta-analysis** (fixed, I^2^ = 0%)		−19.41 [−28.68,-10.14]	<0.0001
Average volume of multiple fibroids	Chen LQ [21]	−1.03 [−1.40, -0.66]	<0.00001
	Gu HH [16]	−0.87 [−1.32, -0.42]	0.0001
	Xiong DM [24]	−1.06 [−1.48, -0.64]	<0.00001
**Subgroup meta-analysis** (fixed, I^2^ = 0%)		−1.00 [−1.23, -0.76]	<0.00001
Volume of fibroids without clarified parameters	Deng XL [22]	−0.68 [−2.38, 1.02]	0.43
	Li LJ [40]	−2.00 [−16.17, 12.17]	0.78
	Luan F [20]	−0.68 [−1.95, 0.59]	0.29
	Mao CX [41]	−0.85 [−1.09, -0.61]	<0.00001
	Mao XG [23]	−7.10 [−10.14, -4.06]	<0.00001
	Wang JY [42]	−5.40 [−8.73, -2.07]	0.001
	Wang XR [18]	−1.35 [−1.77, -0.93]	<0.00001
	Wu JH [43]	−3.10 [−4.18, -2.02]	<0.00001
	Ying LJ [25]	−1.24 [−2.55, 0.07]	0.06
	Zhang LY [26]	−1.11 [−2.46, 0.24]	0.11

^a^Gao CR with three arms had two comparisons. ^b^Hu WH a: Guizhi Fuling capsules were taken 3# three times daily for 6 months; ^c^Hu WH b: Guizhi Fuling capsules were taken 5# three times daily for 2 months and 3# three times daily for 4 months.

##### Guizhi Fuling Formula versus mifepristone

Five trials in different subgroups reported the volume of uterine fibroids. In a three-arm trial [15], increased and common dosages of Guizhi Fuling capsules were designed to compare with mifepristone. No significant difference was found between the common dosage of Guizhi Fuling capsules and mifepristone on reducing volume of fibroids (*p* = 0.22). But when the dosage of Guizhi Fuling capsules was increased, fibroid volume decreased as compared with mifepristone (*p* < 0.00001). One trial [31] showed mifepristone had better effect in reducing volume of fibroids (*p* = 0.01). One trial [27] reported no significant difference between Guizhi Fuling Formula and mifepristone in reducing average diameter of fibroids (cm) (MD −2.20, 95% CI −4.55 to 0.15, p = 0.07).

##### Guizhi Fuling Formula versus other Chinese patent medicine

Two trials [28,44] reported number of participants with shrinkage of fibroids based on different standards. One trial [28] defined volume reduction ratio of fibroids ≥ 80% as markedly effective (RR 1.37, 95% CI 0.54 to 3.45, *p* = 0.50), and defined 21% < volume reduction ratio of fibroids < 80% as effective (RR 0.99, 95% CI 0.75 to 1.29, *p* = 0.93). Another trial [44] defined volume reduction ratio of fibroids ≥ 50% as effective (RR 0.96, 95% CI 0.79 to 1.17, *p* = 0.69).

##### Guizhi Fuling Formula plus western medicine versus western medicine

Twenty eight trials are in this comparison, and they applied different parameters to represent the volume of uterine fibroids.

Eight trials reported average volume of maximum fibroids, and the heterogeneity is significant (I^2^ = 93%, random, *P* < 0.00001) (Additional file 3), which may due to clinical heterogeneity or low methodological quality.

Pooling results of two trials [14,39] (Additional file 4) that reported total volume of multiple fibroids and three trials [16,21,24] (Additional file 5) that reported average volume of multiple fibroids, favored Guizhi Fuling Formula plus mifepristone in reducing volume of fibroids.

Ten trials were in the subgroup of volume of fibroids without clarified parameters, Of which five trials [18,23,41-43] showed that Guizhi Fuling Formula plus mifepristone had better effect on reducing volume of fibroids.

One trial [17] reported the volume change of fibroids from baseline, favoring Guizhi Fuling Formula plus mifepristone in reducing volume of fibroids (MD 6.39, 95% CI 5.93 to 6.85, *p* < 0.00001). One trial [45] comparing Guizhi Fuling Formula plus testosterone propionate versus testosterone propionate alone showed no significant difference on reducing fibroid volume (MD −1.45, 95% CI −4.09 to 1.19, *p* = 0.28). One trial [46] reported average diameter of fibroids, showing Guizhi Fuling Formula plus mifepristone had better effect on reducing diameter of fibroids (MD −0.22, 95% CI −0.33 to −0.11, *p* = 0.0001). Two trials [47,48] reported number of participants with shrinkage of fibroids based on different standards. One of the two trials [47] defined volume reduction ratio of fibroids ≥ 60% as markedly effective (RR 1.47, 95% CI 1.10 to 1.96, *p* = 0.009), and defined 20% ≤ volume reduction ratio of fibroids < 60% as effective (RR 0.73, 95% CI 0.41 to 1.29, *p* = 0.27). The other trial [48] defined volume reduction ratio of fibroids ≥ 30% as markedly effective (RR 1.44, 95% CI 1.00 to 2.10, *p* = 0.05), and defined 10% < volume reduction ratio of fibroids < 30% as effective (RR 0.88, 95% CI 0.61 to 1.28, *p* = 0.51).

#### Improvement of fibroids-related symptoms

Five trials [15,21,24,31,44] reported the number of participants whose fibroid-related symptoms were improved (Table 2). Data of the five trials could not be pooled due to ambiguous definition of improvement of symptoms.

**Table 2:** Effect estimate of the number of participants with improvement of symptoms

**Symptoms**	**Study ID**	**RR [95% CI]**	***P *** **value**
**Guizhi Fuling Formula versus mifepristone**			
Heavy menstrual bleeding	^a^Hu WH a [15]	1.20 [0.63, 2.29]	0.58
	^b^Hu WH b [15]	1.25 [0.67, 2.32]	0.48
	Xiong DM [31]	1.09 [0.89, 1.34]	0.42
Prolonged menstrual bleeding	Hu WH a [15]	1.05 [0.77, 1.44]	0.76
	Hu WH b [15]	1.03 [0.76, 1.40]	0.86
	Xiong DM [31]	1.10 [0.92, 1.30]	0.30
Vaginal bleeding at times other than menstruation	Hu WH a [15]	1.20 [0.63, 2.29]	0.58
	Hu WH b [15]	1.25 [0.67, 2.32]	0.48
	Xiong DM [31]	2.00 [0.37, 10.92]	0.42
Distention pain of lower back and abdomen	Hu WH a [15]	1.10 [0.79, 1.55]	0.57
	Hu WH b [15]	1.18 [0.88, 1.58]	0.27
	Xiong DM [31]	1.20 [0.77, 1.87]	0.41
Dysmenorrhea	Hu WH a [15]	1.16 [0.77, 1.74]	0.48
	Hu WH b [15]	1.21 [0.84, 1.75]	0.30
	Xiong DM [31]	2.27 [1.04, 4.97]	0.04
**Guizhi Fuling Formula versus other Chinese patent medicine**			
Prolonged menstrual bleeding	Long X [44]	0.91 [0.73, 1.13]	0.38
Heavy menstrual bleeding	Long X [44]	0.85 [0.67, 1.10]	0.22
Distention of lower abdomen	Long X [44]	0.96 [0.76, 1.21]	0.73
**Guizhi Fuling Formula plus mifepristone versus mifepristone**			
Heavy menstrual bleeding	Chen LQ [21]	1.11 [0.94, 1.31]	0.24
	Xiong DM [24]	1.13 [0.93, 1.37]	0.22
Prolonged menstrual bleeding	Chen LQ [21]	1.05 [0.89, 1.23]	0.56
	Xiong DM [24]	1.10 [0.92, 1.30]	0.30
Vaginal bleeding at times other than menstruation	Chen LQ [21]	1.60 [0.55, 4.68]	0.39
	Xiong DM [24]	1.13 [0.42, 3.00]	0.81
Distention pain of lower back and abdomen	Chen LQ [21]	1.60 [0.95, 2.68]	0.08
	Xiong DM [24]	1.74 [0.98, 3.09]	0.06
Dysmenorrhea	Chen LQ [21]	1.31 [0.92, 1.86]	0.14
	Xiong DM [24]	2.35 [1.15, 4.82]	0.02

^a^Hu WH a: Guizhi Fuling capsules were taken 3# three times daily for 6 months; ^b^Hu WH b: Guizhi Fuling capsules were taken 5# three times daily for 2 months and 3# three times daily for 4 months.

##### Guizhi Fuling Formula versus mifepristone

Guizhi Fuling Formula was found in one trial [31] to improve dysmenorrhea symptoms over mifepristone.

##### Guizhi Fuling Formula versus other Chinese patent medicine

One trial [44] showed no significant difference between Guizhi Fuling Formula and another Chinese patent medicine in number of participants with improvement of fibroid-related symptoms.

##### Guizhi Fuling Formula plus mifepristone versus mifepristone

One trial [24] favored Guizhi Fuling Formula plus mifepristone in number of participants with improvement of dysmenorrhea.

#### Volume of uterus

Twelve trials reported the volume of uterus.

##### Guizhi Fuling Formula versus mifepristone

One trial [14] reported there was no significant difference between Guizhi Fuling Formula and mifepristone on reducing volume of uterus (MD 17.00, 95% CI −9.98 to 43.98, *p* = 0.22). Another trial [49] reported total volume of uterus and fibroids, and found mifepristone was superior to Guizhi Fuling Formula in reducing total volume of uterus and fibroids (MD 112.52, 95% CI 88.52 to 136.52, *p* < 0.00001).

##### Guizhi Fuling Formula versus other Chinese patent medicines

One trial [13] reported the average of the sum of three diameters of uterus, finding no significant difference between Guizhi Fuling Formula and other Chinese patent medicines in reducing diameters (MD −0.22, 95%CI −0.91 to 0.47, *p* = 0.53).

##### Guizhi Fuling Formula plus mifepristone versus mifepristone

Nine trials [14,19,20,22,25,26,34,42],[43] reported uterine volume (Table 3).

**Table 3:** **Effect estimate of Guizhi Fuling Formula for volume of uterus (cm3)**^**a**^

**Study ID**	**MD [95% CI]**	***P *** **value**
Deng XL [22]	−15.80 [−29.29, -2.31]	0.02
Gao CR [14]	−100.00 [−125.53, -74.47]	<0.00001
Luan F [20]	−15.80 [−28.54, -3.06]	0.02
Teng MJ [34]	−74.75 [−86.45, -63.05]	<0.00001
Wang JY [42]	−11.00 [−16.04, -5.96]	<0.0001
Wu JH [43]	−23.50 [−34.26, -12.74]	<0.0001
Ying LJ [25]	−28.80 [−47.82, -9.78]	0.003
Zhang LY [26]	−18.38 [−30.15, -6.61]	0.002
Zhao YF [19]	−23.16 [−36.41, -9.91]	0.0006
**Meta-Analysis** (random,I^2^ = 94%)	−33.14 [−48.92, -17.36]	<0.0001

^a^Comparison for this outcome is Guizhi Fuling Formula plus mifepristone versus mifepristone.

Even when random-effect model was applied, heterogeneity was too large (I^2^ = 94%), which may have been due to clinical heterogeneity or low methodological quality. We explored the existence of heterogeneity. When we excluded two trials [14,34] with outliers of larger volume of uterus before treatment, the pooling data showed Guizhi Fuling Formula plus mifepristone had better effect on reducing uterine volume than mifepristone alone (MD −15.45, 95%CI −19.09 to −11.81, I^2^ =30%, fixed, *p* < 0.00001). So we considered that heterogeneity may have resulted from the particularity of included participants.

#### Safety

No trial reported serious adverse events. Twenty-three trials (60.53%, 23/38) did not report information about adverse events. Fifteen trials (39.47%, 15/38) reported adverse events, including nausea, vomiting, gastrointestinal discomfort, poor appetite, stomachache, distending pain of the breasts, sensation of bearing down and expansion in the anal region, weakness, sleepiness, hyperhidrosis, hectic fever, vaginal bleeding at times other than menstruation, low libido, dizziness, headache, palpitation, abdominal pain or abdominal distension, impaired liver function, and pruritus. Thirteen trials (34.21%, 13/38) reported the number of participants who experienced adverse events.

Meta-analysis of two trials [27,30] comparing Guizhi Fuling Formula with mifepristone showed Guizhi Fuling Formula was potentially safer than mifepristone based on the incidence of adverse events (RR 0.42, 95% CI 0.24 to 0.75, *p* = 0.003, I^2^ = 46%, fixed).

Meta-analysis of 11 trials [17,21,23-25,29,38,41,46,48],[50] comparing Guizhi Fuling Formula plus mifepristone with mifepristone alone showed Guizhi Fuling Formula plus mifepristone might experience less adverse events than mifepristone alone (RR 0.69, 95% CI 0.52 to 0.91, *p* = 0.01, I^2^ = 45%, fixed), indicating that Guizhi Fuling Formula might offset some adverse events in the combination with western medicine.

## Discussion

### Main findings

Twenty-one trials in our study (three meta-analyses and eight individual trials) showed that Guizhi Fuling Formula plus mifepristone may be more effective than mifepristone alone in reducing the volume of fibroids. Nine trials (one meta-analysis) showed that Guizhi Fuling Formula plus mifepristone may be more effective than mifepristone alone in reducing the volume of the uterus. The two findings were consistent with the Cochrane review [11], but our systematic review included more trials. The difference of the number of trials between the Cochrane review and our review could be accounted for by the newer search, different search strategies, and inclusion/exclusion criteria.

We also found that one trial [31] favored Guizhi Fuling Formula over mifepristone alone with regard to the number of participants with improvement of dysmenorrhea. Another trial [24] found Guizhi Fuling Formula plus mifepristone had better effect than mifepristone on number of participants with improvement of dysmenorrhea. Two trials (one meta-analysis) [27,30] showed Guizhi Fuling Formula was potentially safer than mifepristone, and 11 trials (one meta-analysis) showed Guizhi Fuling Formula plus mifepristone resulted in fewer adverse events than mifepristone alone.

### Strengths and limitations

There are several strengths in this study. This is a systematic review and meta-analysis on an important topic of women health. The search for eligible trials was comprehensive and most of the included RCTs identified in Chinese databases were not found in English databases. We had a standard protocol registration in PROSPERO, an international prospective register of systematic reviews, and published the protocol. Moreover, we rigorously assessed the methodological quality of included trials.

Our study also has several limitations. The methodological quality of the included trials was generally poor. The majority of trials did not report randomization procedures and all trials lacked information on blinding. Additionally, intention-to-treat analysis and pre-trial sample size estimate were not applied. We failed to perform a funnel plot analysis due to insufficient number of included trials in meta-analysis, so there may be potential publication bias. Two meta-analyses were limited in performance due to very large unexplained heterogeneity. Though we endeavored to contact trial authors to clarify the missing information, responses were not satisfactory. We tried to compare Guizhi Fuling Formula with other Chinese patent medicines, but failed to draw any conclusions due to the limited number of such trials.

### Interpretation

The mechanism of Guizhi Fuling Formula to shrink or eliminate fibroids and improve clinical manifestations might be as follows: Guizhi Fuling Formula inhibits tumor growth, angiopoiesis [51] and inflammation [52]. In terms of Chinese medicine actions, the formula invigorates blood, dissolves stasis, and resolves masses [9].

### Implications for further study

Most of included trials lacked detailed information on methodology, since the reporting of most RCTs published in the Chinese literature did not follow the CONSORT statement. Furthermore, the low number of telephone or e-mail responses of original authors did not provide some of the missing information we needed. To improve methodological quality of clinical trials, we suggest the following issues should be addressed: randomisation methods need to be described clearly and reported fully; double blinding should be carried out with the use of adequate placebo, if possible, although blinding of participants and practitioners may be difficult for different traditional Chinese preparations, at the very least every attempt should be made to blind outcome assessors and statisticians; withdrawal/dropout during the trial and use of intention-to-treat analysis should be clearly described; pre-trial sample size should be estimated; the protocol should be registered and reported as a link in the article.

For outcome measures, fibroid or uterine volume and clinically relevant outcomes are important for clinical research on uterine fibroids. Volume can be determined by measuring length, height, and width, and calculated with the equation for a prolate ellipse using π/6(0.523) × length × width × height [53]. Although there is no standard in reporting fibroid volume, the volume of the maximum fibroid could be a good choice as it is easier to carry out and is more accurate. Clinically relevant outcomes, such as fibroid-related symptoms, quality of life, should be addressed and measured using validated instruments, such as a pictorial blood-loss assessment chart, Short-Form McGill Pain Questionnaire or visual-analogue scale for pain, and the uterine fibroid symptoms quality of life (UFS-QOL) questionnaire. In addition, composite outcomes are not preferred and we recommend each outcome should be reported individually with clear definition or judgment. Finally, future trials should pay more attention to adverse events, especially long-term safety of the therapy being investigated. Adverse events should be recorded and reported completely, using international standard medical terms.

## Conclusions

The findings of the current trials suggest that Guizhi Fuling Formula plus mifepristone may be more effective than mifepristone alone for the treatment of uterine fibroids in reducing the volume of fibroids or uterus. However, due to poor methodological quality, we could not draw confirmative conclusions on the beneficial effect of Guizhi Fuling Formula plus mifepristone for uterine fibroids. We recommend that further well-designed clinical trials with large sample sizes be undertaken.

## Competing interests

The authors declare that they have no competing interests.

## Authors’ contributions

JPL obtained funding for the study, conceived and designed the study. NNC conducted study search and identification with MH, and conducted inclusion/exclusion, study selection, data extraction, quality assessment with JPL and MH. NNC wrote the first draft of the manuscript. GYY contributed to English writing and manuscript revision. YYW contributed to data analysis. NNC, JPL, MH, HY and XKW participated in the revision of subsequent draft. All authors read and approved the final manuscript.

## Pre-publication history

The pre-publication history for this paper can be accessed here:

http://www.biomedcentral.com/1472-6882/14/2/prepub

## Supplementary Material

Additional file 1 Characteristics of included studies. Click here for file

Additional file 2 Risk of bias of included studies. Click here for file

Additional file 3 Guizhi Fuling Formula plus mifepristone versus mifepristone for average volume of maximum fibroids. Click here for file

Additional file 4 Guizhi Fuling Formula plus mifepristone versus mifepristone for total volume of multiple fibroids. Click here for file

Additional file 5 Guizhi Fuling Formula plus mifepristone versus mifepristone for average volume of multiple fibroids. Click here for file
