# Supplementary material for: Chinese herbal medicine Guizhi Fuling Formula for treatment of uterine fibroids: a systematic review of randomised clinical trials
Source: BMC Complement Altern Med. 2014 Jan 2;14:2. doi: 10.1186/1472-6882-14-2 (PMC3881498; doi:10.1186/1472-6882-14-2)
Supplement: Additional file 1 — Characteristics of included studies. [file 1472-6882-14-2-S1.docx]

**Characteristics of included studies**

| **Study ID** | **Sample**  **size** | **Age (y) (T/C)** | **Average volume of fibroids (cm^3^) (T/C)** | **Intervention** | **Control** | **Duration**  **(mo)** | **Follow- up (mo)** | **Outcome measures** |
| --- | --- | --- | --- | --- | --- | --- | --- | --- |
| Chen LQ 2008 [21] | 70 | 36.4±4.2/  38.3±5.1 | 5.41±1.15/  5.68±1.04 | Guizhi Fuling capsules + mifepristone (4# three times daily + 12.5 mg once daily) | Mifepristone  (12.5 mg once daily) | 3 | 6 | Volume of fibroids, symptoms, adverse events |
| Chen PM 2001 [49]^a^ | 54 | 39.8±7.1/  40.3±8.7 | NA | Guizhi Fuling capsules (2# three times daily) | Mifepristone (25 mg once daily ) | 3 | 12 | Volume of uterus |
| Chen XJ 2008 [32] | 136 | NA | 7.87±1.85  /8.46±1.14 | Guizhi Fuling capsules + mifepristone (4# three times daily + 25 mg once daily) | Mifepristone  (25 mg once daily) | 3 | 3 | Volume of fibroids |
| Deng XL 2010 [22] | 69 | 38.9±8.1/  39.5±8.9 | 12.88±7.75/  12.42±7.53 | Guizhi Fuling capsules + mifepristone (3# three times daily + 12.5 mg once daily) | Mifepristone  (12.5 mg once daily) | 3 | 12-24 | Volume of fibroids, volume of uterus |
| Feng FQ 2003 [27] | 178 | 42.7±4.1 | 47.6±12.7/  46.9±12.6  (diameter) | Guizhi Fuling capsules (3# three times daily) | Mifepristone  (12.5 mg once daily) | 3 | 24 | Volume of fibroids, adverse events |
| Gao CR 2012b [14] | 40 | 38.3±7.1/  40.3±10.1 | 80±21/86±20 | Guizhi Fuling capsules (3# three times daily) | Mifepristone (12.5 mg once daily) | 3 | 6 | Volume of fibroids, volume of uterus, recurrence rate |
| Gao CR 2012a [14] | 40 | 37.4±5.1/  40.3±10.1 | 83±24/86±20 | Guizhi Fuling capsules + mifepristone (3# three times daily + 12.5 mg once daily) | Mifepristone (12.5 mg once daily) | 3 | 6 | Volume of fibroids, volume of uterus, recurrence rate |
| Gu HH 2011 [16] | 134 | 34.2±7.2/  35.5±6.6 | 8.51±1.31/  8.42±1.50 | Guizhi Fuling capsules + mifepristone (4# three times daily + 12.5 mg once daily) | Mifepristone  (12.5 mg once daily) | 3 | NA | Volume of fibroids |
| Gu Y 2012 [33] | 80 | NA | 8.02±1.68/  8.36±1.15 | Guizhi Fuling capsules + mifepristone (4# three times daily + 25 mg once daily) | Mifepristone  (25 mg once daily) | 3 | 3 | Volume of fibroids |
| Hu WH 2009b [15] | 80 | 37±5.47 | 5.31±1.52/  5.19±1.49 | Guizhi Fuling capsules (5# three times daily for two months→3# three times daily for four months) | Mifepristone  (12.5 mg once daily) | 6 | NA | Volume of fibroids, symptoms |
| Hu WH 2009a [15] | 64 | 37±5.47 | 5.09±1.21/  5.19±1.49 | Guizhi Fuling capsules (3# three times daily) | Mifepristone  (12.5 mg once daily) | 6 | NA | Volume of fibroids, symptoms |
| Jiao JF 2011 [50] | 39 | 45.1/45.5 | NA | Guizhi Fuling capsules + mifepristone (3# three times daily + 12.5 mg once daily) | Mifepristone  (12.5 mg once daily) | 3 | 6 | Adverse events |
| Li LJ 2009 [40] | 90 | 45.7±8.9/  46.1±9.2 | 42.2±8.3/  39.8±9.1 | Guizhi Fuling capsules + mifepristone (3# three times daily + 25 mg once daily) | Mifepristone (25 mg once daily) | 3 | NA | Volume of fibroids |
| Liu SQ 2013 [17] | 116 | 42.87±2.53/  43.17±2.42 | 23.72±2.78/  24.12±2.80 | Guizhi Fuling capsules + mifepristone (3# three times daily + 12.5 mg once daily) | Mifepristone (12.5 mg once daily) | 3 | NA | Volume of fibroids, recurrence rate, adverse events |
| Long X 2011 [44]^a^ | 113 | 30.3±7.1 | NA | Guizhi Fuling capsules (3# three times daily) | Gongliuning capsules (6# three times daily) | 3 | NA | Volume of fibroids, symptoms |
| Luan F 2006 [20] | 138 | NA | 11.98±7.53/  11.52±7.31 | Guizhi Fuling capsules + mifepristone (3# three times daily + 12.5 mg once daily) | Mifepristone (12.5 mg once daily) | 3 | 12-24 | Volume of fibroids, volume of uterus |
| Lu HJ 2010 [39] | 120 | NA | 111.6±37.0/  110.6±36.8 | Guizhi Fuling capsules + mifepristone (3# three times daily + 12.5 mg once daily) | Mifepristone (12.5 mg once daily) | 3 | NA | Volume of fibroids |
| Luo LY 2004 [28] | 126 | 37.5/37 | NA | Guizhi Fuling capsules (3# three times daily) | Gongliuqing capsules (3# three times daily) | 3 | 2 | Volume of fibroids |
| Luo XQ 2012 [46] | 78 | 43.38±4.69/  43.33±4.78 | 3.93±0.87/  2.89±0.87  (diameter) | Guizhi Fuling capsules + mifepristone (4# three times daily + 10mg once daily) | Mifepristone (10 mg once daily) | 3 | NA | Volume of fibroids, adverse events |
| Mao CX 2012 [41] | 120 | 36.5±6.5/  36.0±7.5 | 5.52±1.09/  5.49±1.24 | Guizhi Fuling capsules + mifepristone (3# three times daily + 12.5 mg once daily) | Mifepristone (12.5 mg once daily) | 3 | 3 | Volume of fibroids, adverse events |
| Mao XG 2012 [23] | 66 | 43.6±4.8 | NA | Guizhi Fuling pills + mifepristone (3# three times daily + 25 mg twice daily) | Mifepristone (25 mg twice daily) | 3 | 12 | Volume of fibroids, recurrence rate, adverse events |
| Shen D 2006 [45] | 40 | 41.02±5.33/  41.03±4.82 | 12.88±6.9/  12.3±6.96 | Guizhi Fuling capsules + testosterone propionate (3# three times daily + 25 mg intramuscular injection once daily for 3 days from day 1 of menstruation, and then 25 mg once weekly) | Testosterone propionate (25 mg intramuscular injection once daily for 3 days from day 1 of menstruation, and then 25 mg once weekly) | 3 | NA | Volume of fibroids |
| Teng MJ 2007 [34] | 82 | 27-51/26-50 | 95.83±12.91/  84.59±11.22 | Guizhi Fuling capsules + mifepristone (3# three times daily + 25mg once daily) | Mifepristone (25 mg once daily) | 3 | NA | Volume of fibroids, volume of uterus |
| Wang DQ 2012 [29] | 76 | 31.25±10.63/  32.36±10.05 | NA | Guizhi Fuling capsules + mifepristone (3# three times daily + 12.5 mg once daily) | Mifepristone (12.5 mg once daily) | 3 | NA | Adverse events |
| Wang JY 2011 [42] | 100 | 36 | 56.4±11.2/  58.3±10.5 | Guizhi Fuling pills + mifepristone (6 g once daily + 12.5 mg once daily) | Mifepristone (12.5 mg once daily) | 3 | NA | Volume of fibroids, volume of uterus |
| Wang XR 2011 [18] | 120 | 35.8±6.7/  37.1±6.3 | 8.28±1.44/  8.55±1.61 | Guizhi Fuling capsules + mifepristone (4# three times daily + 12.5 mg once daily) | Mifepristone (12.5 mg once daily) | 3 | NA | Volume of fibroids |
| Wang YL 2004 [47] | 126 | 47/46 | NA | Guizhi Fuling capsules + mifepristone (3# three times daily + 12.5mg once daily) | Mifepristone (12.5 mg once daily) | 6 | 6 | Volume of fibroids |
| Wei LH 2010 [13]^a^ | 195 | NA | NA | Guizhi Fuling capsules (3# three times daily) | Gongliuxiao capsules (3# three times daily) | 3 | NA | Volume of uterus, adverse events |
| Wu C 2012 [48] | 150 | 43.0±2.3/  44.0±2.5 | NA | Guizhi Fuling capsules + mifepristone (3# three times daily + 12.5 mg once daily) | Mifepristone (12.5 mg once daily) | NA | NA | Volume of fibroids, adverse events |
| Wu JH 2011 [43] | 102 | 45.6±9.7/  46.8±10.4 | 12.9±5.6/  12.7±5.4 | Guizhi Fuling capsules + mifepristone (4# three times daily + 10 mg once daily) | Mifepristone (10 mg once daily) | 3 | NA | Volume of fibroids, volume of uterus |
| Wu YF 2011 [35] | 76 | 42.3±3.6/  39.1±5.8 | 32.68±11.07/  34.13±12.3 | Guizhi Fuling capsules + mifepristone (4# three times daily + 25 mg once daily) | Mifepristone (25 mg once daily) | 3 | NA | Volume of fibroids |
| Xiong DM 2006 [31] | 76 | 37.6±4.1/  49.3±9.1 | 5.78±1.15/  5.89±0.97 | Guizhi Fuling capsules (4# three times daily) | Mifepristone (12.5 mg once daily) | 6 | 3 | Volume of fibroids, symptoms |
| Xiong DM 2006a [24] | 68 | 37.4±5.1  40.3±10.1 | 5.52±1.05/  5.98±0.94 | Guizhi Fuling capsules + mifepristone (4# three times daily + 12.5 mg once daily) | Mifepristone (12.5 mg once daily) | 3 | 6 | Volume of fibroids, symptoms, adverse events |
| Yang ZQ 2008 [36] | 122 | NA | 27.87±1.74/  28.36±1.04 | Guizhi Fuling capsules + mifepristone (4# three times daily + 10 mg once daily) | Mifepristone (10 mg once daily) | 3 | 3 | Volume of fibroids |
| Ying LJ 2012 [25] | 70 | 39.3±6.7/  40.1±7.0 | 11.88±6.28/  11.91±6.55 | Guizhi Fuling capsules + mifepristone (3# three times daily + 12.5 mg once daily) | Mifepristone (12.5 mg once daily) | 3 | NA | Volume of fibroids, volume of uterus, adverse events |
| Yue Li 2013 [37] | 82 | 40.3±3.5/  37.2±4.7 | 31.98±10.06/  32.48±11.30 | Guizhi Fuling capsules + mifepristone (4# three times daily + 10 mg once daily) | Mifepristone (10 mg once daily) | 3 | NA | Volume of fibroids |
| Zhao YF 2013 [19] | 120 | 30-55/30-55 | 25.87±5.52/  26.08±5.33 | Guizhi Fuling capsules + mifepristone (3# three times daily + 12.5 mg once daily) | Mifepristone (12.5 mg once daily) | 3 | 12-24 | Volume of fibroids, volume of uterus, adverse events |
| Zhang LY 2010 [26] | 142 | 36.3±2.4 | 12.27±6.87/  12.26±6.52 | Guizhi Fuling capsules + mifepristone (3# three times daily + 12.5 mg once daily) | Mifepristone (12.5 mg once daily) | 3 | 12-24 | Volume of fibroids, volume of uterus |
| Zhong GP 2012 [38] | 88 | 42.9±5.2 | NA | Guizhi Fuling capsules + mifepristone (4# three times daily + 25 mg once daily) | Mifepristone (25 mg once daily) | 3 | 12 | Volume of fibroids, adverse events |
| Zhu YJ 2009 [30] | 79 | 41.20/41.03 | 12.45±6.6/  12.30±6.86 | Guizhi Fuling pills (6# once daily) | Mifepristone (5 mg once daily ) | 3 | 6-12 | Volume of fibroids, volume of uterus, adverse events |

^a^Trial applied Guizhi Fuling formula for participants in control group.
