# Supplementary material for: Chinese herbal medicine Guizhi Fuling Formula for treatment of uterine fibroids: a systematic review of randomised clinical trials
Source: BMC Complement Altern Med. 2014 Jan 2;14:2. doi: 10.1186/1472-6882-14-2 (PMC3881498; doi:10.1186/1472-6882-14-2)
Supplement: Additional file 2 — Risk of bias of included studies. [file 1472-6882-14-2-S2.docx]

**Risk of bias of included studies**

| Study ID | Random sequence generation | Allocation concealment | Blinding of participants and personnel | Blinding of outcome assessment | Incomplete outcome data | Selective reporting | ^a^Other bias |
| --- | --- | --- | --- | --- | --- | --- | --- |
| Chen LQ 2008 [21] | Unclear | Unclear | High | Unclear | Unclear | High | Unclear |
| Chen PM 2011 [49] | Unclear | Unclear | High | Unclear | Unclear | Low | Unclear |
| Chen XJ 2008 [32] | Unclear | Unclear | High | Unclear | Unclear | Low | Unclear |
| Deng XL 2010 [22] | Unclear | Unclear | High | Unclear | Unclear | High | Unclear |
| Feng FQ 2003 [27] | Unclear | Unclear | High | Unclear | Unclear | Unclear | High |
| Gao CR 2012 [14] | Unclear | Unclear | High | Unclear | Unclear | Low | Unclear |
| Gu HH 2011 [16] | Unclear | Unclear | High | Unclear | Unclear | Low | Unclear |
| Gu Y 2012 [33] | Unclear | Unclear | High | Unclear | Unclear | Low | Unclear |
| Hu WH 2009 [15] | Unclear | Unclear | High | Unclear | High | Low | Unclear |
| Jiao JF 2011 [50] | Unclear | Unclear | High | Unclear | Unclear | Low | Unclear |
| Li LJ 2009 [40] | Unclear | Unclear | High | Unclear | Unclear | Low | Unclear |
| Liu SQ 2013 [17] | Unclear | Unclear | High | Unclear | Unclear | Unclear | Unclear |
| Long X 2011 [44] | Unclear | Unclear | High | Unclear | Unclear | Low | High |
| Luan F 2006 [20] | Unclear | Unclear | High | Unclear | Low | High | High |
| Lu HJ 2010 [39] | Unclear | Unclear | High | Unclear | Unclear | Low | Unclear |
| Luo LY 2004 [28] | Unclear | Unclear | Unclear | Unclear | Unclear | Unclear | Unclear |
| Luo XQ 2012 [46] | Unclear | Unclear | High | Unclear | Unclear | Low | Unclear |
| Mao CX 2012 [41] | Unclear | Unclear | High | Unclear | Unclear | Low | Unclear |
| Mao XG 2012 [23] | Unclear | Unclear | High | Unclear | Unclear | High | Unclear |
| Shen D 2006 [45] | Unclear | Unclear | High | Unclear | Unclear | Low | Unclear |
| Teng MJ 2007 [34] | Unclear | Unclear | High | Unclear | Unclear | Low | High |
| Wang DQ 2012 [29] | Unclear | Unclear | High | Unclear | Unclear | Unclear | Unclear |
| Wang JY 2011 [42] | Unclear | Unclear | High | Unclear | Unclear | Low | Unclear |
| Wang XR 2011 [18] | Low | Unclear | High | Unclear | Unclear | Low | Unclear |
| Wang YL 2004 [47] | Unclear | Unclear | High | Unclear | Unclear | Low | Unclear |
| Wei LH 2010 [13] | Low | Unclear | Unclear | Unclear | Low | Low | Unclear |
| Wu C 2012 [48] | Unclear | Unclear | High | Unclear | Unclear | Low | Unclear |
| Wu JH 2011 [43] | Unclear | Unclear | High | Unclear | Unclear | Low | Unclear |
| Wu YF 2011 [35] | Unclear | Unclear | High | Unclear | Unclear | Low | Unclear |
| Xiong DM 2006 [31] | Unclear | Unclear | High | Unclear | Unclear | Low | High |
| Xiong DM 2006a [24] | Unclear | Unclear | High | Unclear | Unclear | High | Unclear |
| Yang ZQ 2008 [36] | Unclear | Unclear | High | Unclear | Unclear | Low | Unclear |
| Ying LJ 2012 [25] | Unclear | Unclear | High | Unclear | Unclear | High | Unclear |
| Yue Li 2013 [37] | Unclear | Unclear | High | Unclear | Unclear | Low | Unclear |
| Zhao YF 2013 [19] | Low | Unclear | High | Unclear | Unclear | Low | Unclear |
| Zhang LY 2010 [26] | Unclear | Unclear | High | Unclear | Unclear | High | Unclear |
| Zhong GP 2012 [38] | Unclear | Unclear | High | Unclear | Unclear | Low | Unclear |
| Zhu YJ 2009 [30] | Unclear | Unclear | High | Unclear | Unclear | Low | Unclear |

^a^Other bias refers to sample size estimate, baseline comparability, inclusion and exclusion criteria.
