# Supplementary figures and images for: Chinese herbal medicine Guizhi Fuling Formula for treatment of uterine fibroids: a systematic review of randomised clinical trials
Source: BMC Complement Altern Med. 2014 Jan 2;14:2. doi: 10.1186/1472-6882-14-2 (PMC3881498; doi:10.1186/1472-6882-14-2)

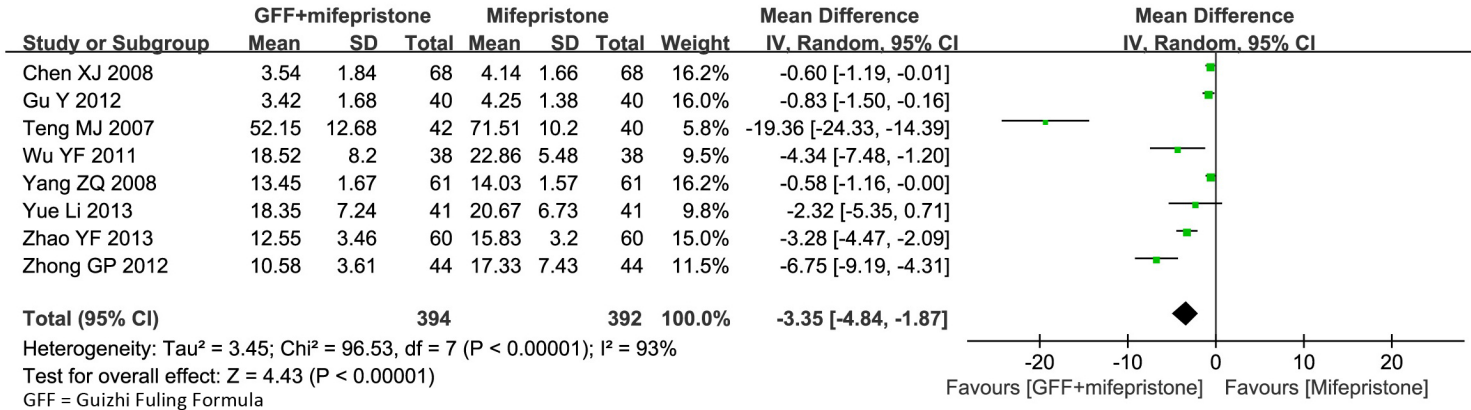

Supplement: Additional file 3 — Guizhi Fuling Formula plus mifepristone versus mifepristone for average volume of maximum fibroids. [file 1472-6882-14-2-S3.pdf]

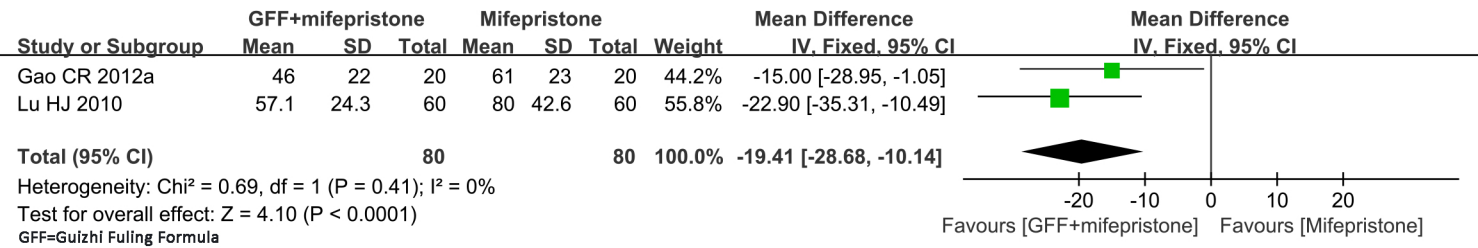

Supplement: Additional file 4 — Guizhi Fuling Formula plus mifepristone versus mifepristone for total volume of multiple fibroids. [file 1472-6882-14-2-S4.pdf]

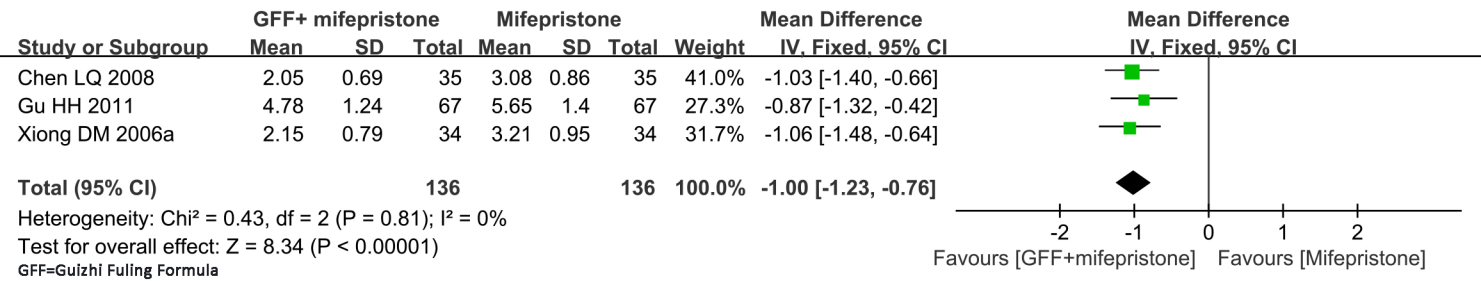

Supplement: Additional file 5 — Guizhi Fuling Formula plus mifepristone versus mifepristone for average volume of multiple fibroids. [file 1472-6882-14-2-S5.pdf]
